# Blinders

**Published:** 1881-09

**Authors:** 


### BLINDERS.

IN the past two years I have occasionally driven, without fear, but with considerable discomfort, a spirited, but kind and affectionate young horse. I have, until recently, used blinders, because other people used them I had no trouble, except when meeting other wagons, or when other wagons came up behind me, and then in spite of all I could do the horse would jump into the ditch, or across it. I observed, however, that he had no fear of objects that he could see distinctly, or of noises that he could account for. It occurred to me that if I were to attempt to walk the length of Broadway with a newspaper directly in front of my eyes, as blinders are in front of a horse's eyes, I should get quite as nervous as my horse ever gets, before I had accomplished the distance, on account of the strange noises and the fear of death or injury from contact with all sorts of objects.

I took off the blinders and threw them away, so that no other horse should suffer from them. I used an open bridle, and in three days I could drive my horse anywhere. He had lost all fear of wagons, or other objects, and a child could drive him with safety. I think that discarding blinders has increased his cash value one hundred per cent.

MANY persons, when they come in from the rain, put their umbrellas in the rack with the handle upward. They should put it downward, because when the handle is upward the water runs down inside to the place to where the ribs are joined to the handle, and cannot get out, but stays, rotting the cloth and rusting the metal until slowly dried away. The wire securing the ribs soon rusts and breaks. If placed the other end up the water readily runs off.

